# Hardware-Related Skin Erosion in Deep Brain Stimulation for Parkinson’s Disease: How Far Can We Go? An Illustrative Case Report

**DOI:** 10.3390/brainsci12121715

**Published:** 2022-12-15

**Authors:** Pietro Zeppa, Marco Fraccalvieri, Chiara Fronda, Enrico Lo Bue, Laura Rizzi, Virginia Caliendo, Michele Maria Lanotte

**Affiliations:** 1 Stereotactic and Functional Neurosurgery Unit, Department of Neuroscience “Rita Levi Montalcini”, AOU Città della Salute e della Scienza di Torino, University of Turin, 10126 Turin, Italy; 2 Plastic Surgery Unit, AOU Città della Salute e della Scienza di Torino, University of Turin, 10126 Turin, Italy; 3 Surgical Dermatology Unit, Department of Surgery, AOU Città della Salute e della Scienza di Torino, 10126 Turin, Italy

**Keywords:** deep brain stimulation, skin erosion, hardware-related complications

## Abstract

Skin erosion is a hardware-related complication commonly described after deep brain stimulation (DBS). Hardware exposure is often associated with the development of infection that can lead to implant removal. However, in selected cases, it is possible to manage skin erosion without having to remove the hardware. This article presents the case of a patient with recurrent skin erosions above the IPG, who underwent multiple surgeries. Given the failure of less invasive approaches, a more complex surgery with the employment of a pedunculated flap of pectoralis major in order to cover the IPG was attempted. Nevertheless, the IPG removal was finally unavoidable, resulting in a rapid decline in clinical performance. This illustrative case suggests how, in patients with sustained stimulation who benefit from a good degree of autonomy, it may be useful to use invasive surgical techniques to resolve skin erosions and save the DBS system. In spite of everything, sometimes complete or partial removal of the implant still becomes unavoidable, but this can lead to a severe worsening of PD symptoms. Definitive removal of the system should therefore be considered only in cases of frank infection or after failure of all other approaches.

## 1. Introduction

Deep brain stimulation (DBS) has become an essential and safe treatment in a variety of movement and neuropsychiatric disorders [1]. Compared to ablative procedures, DBS has the advantage of being modulable and reversible, but introduces potential hardware-related adverse events [2,3]. Hardware complications occur with reported rates ranging from 1.7% to 8.4% per year [2,4]. Among them, skin erosion is one of the most serious and frustrating problems, often associated with the development of a hardware infection that can lead to long-term antibiotic therapy, repeated surgeries, and to the need for hardware removal. Although exposure of the hardware through skin erosion often causes its infection, sometimes, despite prolonged exposure, bacterial colonization does not occur [5]. When obvious signs of infection are associated with skin erosion, surgical debridement and hardware removal is universally recommended [3,6,7]. In cases of non-purulent erosion, there is no consensus on the optimal treatment [8]. Given that the biofilm formed by bacteria on the hardware is very difficult to remove, many authors suggest the removal of the system, even in the case of skin erosion without clear clinical-laboratoristic signs of infection. However, several papers highlight how, in selected cases, it is possible to manage skin erosion without having to remove the hardware, losing all therapeutic benefits [9]. Several articles describe the surgical technique employed to cover skin erosions on the scalp, while the surgical strategy used to cover cutaneous defects above the IPG is not as well described [8,9,10].

In this article, we describe the complex case of a patient with recurrent skin erosions above the IPG (implantable pulse generator), who underwent multiple surgeries. Given the failure of less invasive approaches, a more complex surgery with the employment of a pedunculated flap of pectoralis major muscle in order to cover the IPG was attempted. Nevertheless, IPG removal was finally unavoidable, resulting in a rapid decline in clinical performance.

## 2. Illustrative Case

A 65-year-old patient with idiopathic Parkinson’s disease (PD), no family history of PD, and no relevant comorbidities underwent bilateral DBS of the subthalamic nucleus in 2007, eight years after PD diagnosis, with marked clinical improvement. The IPG was then replaced in May 2013 and March 2017. A year after the last replacement the patient was admitted to our hospital with a wound infection and swab cultures positive for *S. epidermidis*. The IPG was removed, while the extension cables and electrodes were left in place. Cultures of the intraoperative swab collected within the IPG pocket were negative. The patient subsequently underwent antibiotic therapy with rapid resolution of the infection and wound healing. Three months later, a new IPG was implanted. One year later, a small skin erosion (2 cm^2^) developed above the surgical incision of the IPG pocket. Since wound swab cultures were negative, primary wound closure was attempted. Six months later, a new skin erosion developed near the location of the first one. With the aid of a plastic surgeon, after the excision of the compromised skin, the wound was primary closed again without removing the IPG. One year later, a skin erosion of a few centimeters developed cranially, above the extension cables. The skin surrounding the erosion site was not reddened or warm and no other clinical signs of infections was noticed. C-reactive protein (CPR) and white blood cell count were within normal ranges. Swab cultures remained negative. After an initial attempt at conservative treatment, the patient underwent a further revision surgery. A local rotation flap was employed to cover the erosion site and the IPG was replaced.

**Figure 1:**
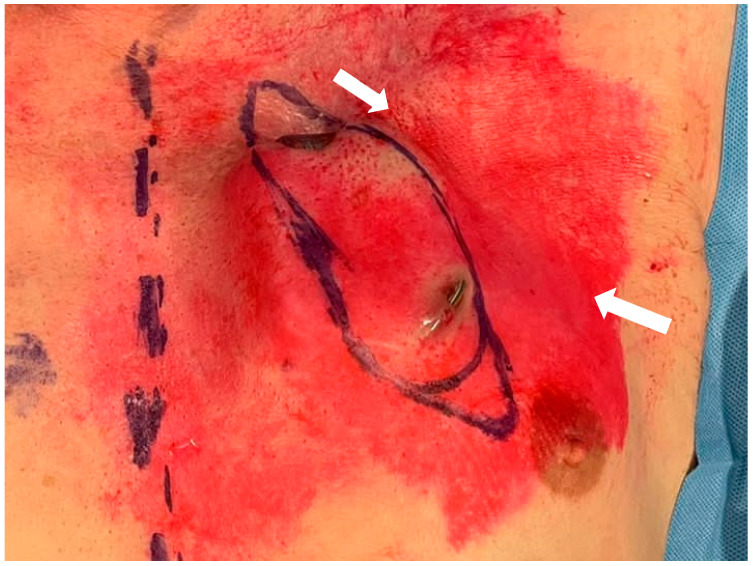
Skin erosions (arrow) above the IPG and above the extension cables. The boundaries of the excised skin portion are drawn.

After six months, the patient was again admitted to our hospital with a skin erosion above the IPG and another one above the extension cables (Figure 1).

**Figure 2:**
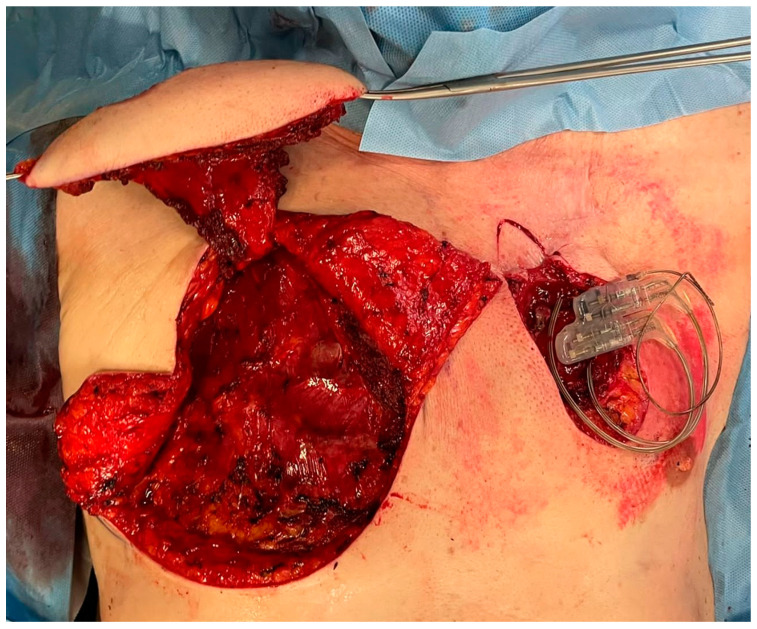
A right pectoralis major myocutaneous flap (PMMF) was prepared after identification of the vascular peduncle.

As in the previous cases, neither clinical nor laboratory evidence of infection was observed. Considering the failures of the previous revision surgeries, after a collegial discussion with the plastic surgeons, a more invasive approach was chosen. The skin overlying the IPG and the caudal portion of extension cables was completely excised up to 1 cm away from the erosion (Figure 1). By blunt dissection, the generator, extension cables, and the previously implanted adapter were isolated. No signs of infection were observed. A swab for culture examination was carried out on the pocket with negative result. A right pectoralis major myocutaneous flap (PMMF) was prepared after identification of the vascular peduncle (Figure 2).

**Figure 3:**
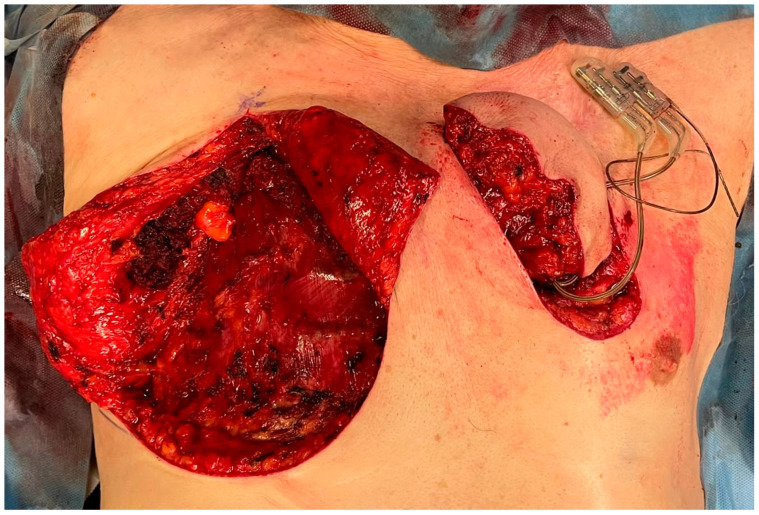
The PMMF was tunneled and brought contralaterally.

The flap was tunneled and brought contralaterally to cover the skin defect (Figure 3).

**Figure 4:**
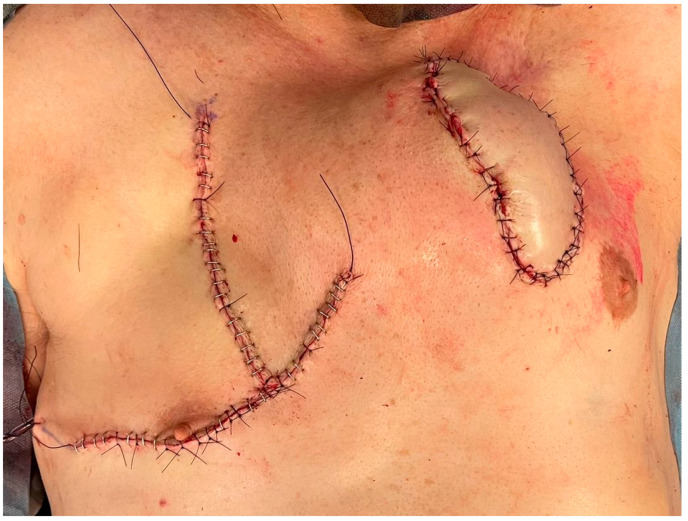
Final wound closure.

Wounds were irrigated with physiological solution. After right pectoral drainage placement, muscular, subcutaneous, and cutaneous planes were sutured (Figure 4).

Impedances were checked and the stimulator was switched on again. Wounds healed regularly. Drainage was removed after 5 days. At 15 days postoperatively, all the stitches were removed.

**Figure 5:**
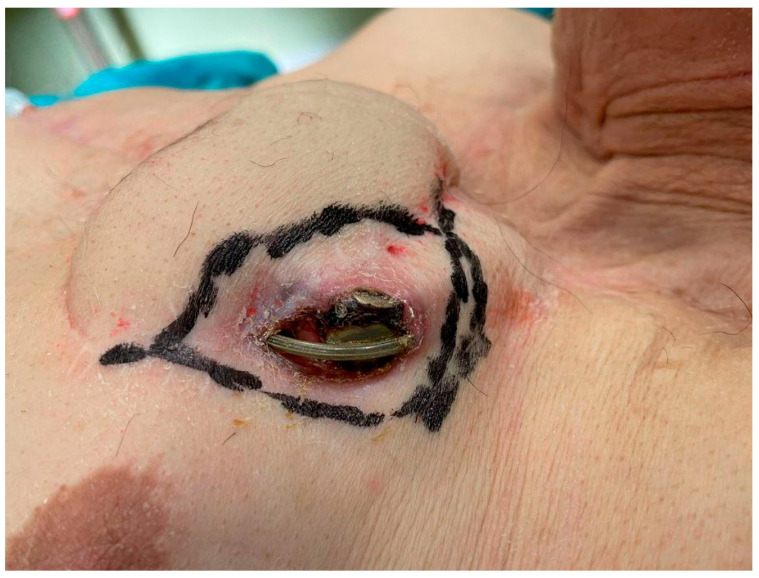
New skin erosion near the surgical scar.

Six months later, the flap was healthy with no signs of infection or further skin erosion. DBS efficacy on Parkinson’s control was preserved. One month later, however, a new erosion developed near the surgical scar of the last surgery with IPG and wire exposure (Figure 5).

Given the failure of even the more complex and invasive surgical approach, it was decided to definitely remove the IPG. The edges of the skin erosion were excised and the wound was closed directly with an interrupted suture. The wound healed regularly, but the patient presented a severe progressive worsening of PD symptoms despite several adjustments of dopaminergic therapy. Currently, five months later, the patient is bedridden in supportive care.

## 3. Discussion

Skin complications after DBS surgery are a major concern for physicians and patients. Their treatment often requires a prolonged course of antibiotics and revision surgeries to either replace components of the DBS system or remove the hardware in its entirety [3]. Reported rates of infection or erosion caused by hardware range from <1% to 24.7% [9]. The most common site of skin erosion is the retromastoid region, where the extension leads are located, followed by the infraclavicular pocket [3,10].

While the exact mechanism of skin erosion in PD patients with DBS has yet to be fully elucidated, the etiology is likely multifactorial and due to hardware, patient characteristics, and surgical factors [11]. PD itself seems to be a risk factor for skin complications, while other risk factors such as hypertension, diabetes, age, gender, disease duration, and disease severity have no influence on the occurrence of skin complications [2,11,12].

Prevention of these complications through refinement of the implantation hardware and surgical techniques are of utmost importance. The most relevant aspects to prevent skin erosion are a tension-free closure and the observance of the vascular supply. The subcutaneous IPG pocket must be adequate; if the pocket is too small the probability of skin erosion is increased due to the tension on the surgical wound by the hardware volume. Similarly, the thickness of subcutaneous tissue overlying the stimulator must be adequate to release tension on the skin, particularly during movement. Moreover, a bulky hardware can be a problem [13]. In order to avoid this, new hardware with a lower profile and smooth edges have been designed to decrease tension over the covering tissue. Despite all the precautions, the risk of skin erosion is unavoidable. Early diagnosis and correct treatment are fundamental for quick recovery.

A small skin erosion without evidence of infection can be at first addressed with a conservative treatment based on wound wash-out and advanced dressings [14]. However in most cases a surgical revision is required [14]. Revision surgery can range from a direct closure to the employment of local, pedicled, or free flaps. Some papers suggest that an initial more aggressive approach can ensure longer-lasting hardware coverage, particularly in the event of major infections or larger erosions [15].

In the illustrative case, a pectoralis major myocutaneous flap (PMMF) was employed in order to cover the erosion site. The PMMF is an ancient flap but always current in the non-microsurgical reconstruction of substance losses in the region of the face and neck, as well as in the anterior region of the thorax [16]. From the point of view of surgical technique, the PMMF can be employed as a flap with a muscle and skin component or with a muscle component only. The use of the PMMF usually results in good coverage of muscle–cutaneous defects, with a low failure rate [17].

Despite all the advantages of using this flap, in the present case it did not prevent new skin erosions and, finally, IPG removal was unavoidable. Nevertheless, the use of the PMMF allowed the removal to be delayed by six months, improving the patient’s quality of life and likely increasing his survival. The rapid decline in the patient’s general condition following the removal of the IPG confirms that all possible efforts had to be attempted to save the implant or at least to delay removal as long as possible.

This illustrative case has the obvious limitation that it cannot be generalized, and no conclusions can be drawn about the effectiveness of using this surgical strategy in managing skin erosions. However, in cases similar to the one described, it might be useful to consider the use of an aggressive surgical approach instead of directly removing the implant. Indeed, the remarkable efficacy of DBS on Parkinson’s disease symptoms is preserved even in the advanced stages of the disease [18]. Although in some cases prolonged survival does not directly translate into increased quality of life, a large proportion of patients treated with DBS can maintain a good degree of autonomy for a long time [19]. The management of any hardware-related complications is therefore of paramount importance to avoid implant removal or at least to delay it as much as possible. To achieve this, collaboration with plastic surgeons and the employment of dedicated surgical approaches, such as the use of pedicled flaps, less familiar to the neurosurgeon, are essential.

## 4. Conclusions

In patients with persistent stimulation benefit and who have retained a good degree of autonomy, it may be useful to use invasive surgical techniques to resolve skin erosions and save the DBS system. Active and early collaboration with plastic surgeons can help to choose the best treatment strategy. In spite of everything sometimes complete or partial removal of the implant still becomes unavoidable, but this can lead to a severe worsening of PD symptoms. Definitive removal of the system should therefore be considered only in cases of frank infection or after failure of all other approaches.

## Data Availability

Not applicable.
